# Functional impairment and serum albumin predict in-hospital mortality in nonagenarians with acute infection: a retrospective cohort study

**DOI:** 10.1186/s12877-019-1301-1

**Published:** 2019-10-15

**Authors:** Wei Huang, Ying Sun, Yunli Xing, Cuiying Wang

**Affiliations:** Department of Geriatrics and Gerontology. Beijing Friendship Hospital, Capital Medical University, No.95 Yong’an Road Xicheng District, Beijing, People’s Republic of China

**Keywords:** Nonagenarian, Acute infection, In-hospital mortality, Serum albumin, Functional impairment

## Abstract

**Background:**

Acute infection leads to substantial mortality in the nonagenarian population. However, the predictive efficacies of functional status and biochemical indexes for in-hospital mortality in these patients remain to be determined.

**Methods:**

A single-center, retrospective cohort study was performed. Consecutive nonagenarian patients who were admitted to our department from January 1, 2014 to December 31, 2016 for acute infectious diseases were included. Baseline data for medical history, functional status, and biochemical indexes were obtained on admission. The outcomes of these patients during hospitalization were recorded. Predictors of in-hospital mortality were identified via logistic regression analyses.

**Results:**

A total of 162 patients were included, and 46 patients died (17.2%) during hospitalization. Univariate analysis showed that the prevalence rates of atrial fibrillation (32.1%) and malignant disease (26.5%) were higher in nonagenarian patients who died during hospitalization than in those who discharged. Multivariate logistic regression analyses identified malignant disease (odds ratio [OR] 2.73, 95% confidence interval [CI]: 1.10–6.78), ADL category (OR 0.82, 95% CI: 0.75–0.89) and serum albumin (OR 0.86, 95%CI 0.78–0.95) as independent predictors of in-hospital mortality in nonagenarian patients hospitalized for acute infection.

**Conclusions:**

Functional impairment as well as serum albumin may be independent predictors of in-hospital mortality in nonagenarian patients hospitalized for acute infectious diseases. Stratification of patients according to Barthel Index score and serum albumin is very necessary.

## Background

The nonagenarian population continues to grow globally with the aging of the population [1]. In developing countries like China, the percentage of people aged 90 and above had reached 0.19% in 2015 with the increase of the average life expectancy from 72.95 years in 2005 to 76.34 years in 2015 [2]. However, these populations are generally excluded from most clinical trials. Due to this lack of evidence in the nonagenarian population, general clinical practice for this population has gained considerable attention during recent years, with clinical trials including nonagenarians mainly focused on the fields of surgery [3–7], chemotherapy [8], interventional operation [9–12] and trauma [13], in the departments of orthopedics, vascular surgery, general surgery, and oncology, rather than a department of internal medicine. In real-world clinical practice, about one-third of the patients over 90 years old have been admitted to an internal medicine department [14]. Moreover, clinical trials studying nonagenarian patients admitted for acute infection, one of the most common causes of hospitalization in these patients [15, 16], have been rarely reported. The clinical characteristics of the nonagenarian patients admitted for acute infection, and more importantly, the predictors of in-hospital mortality in these patients, remain to be determined. Therefore, we performed a cohort study of the nonagenarian patients admitted to the internal medicine department to explore the clinical characteristics of these patients and identify potential determinants of their clinical outcomes.

## Methods

### Participants

All nonagenarians suffering from acute infectious diseases admitted to the Internal Medicine Department of our hospital were consecutively enrolled from January 1, 2014 to December 31, 2016. Our hospital is one of the tertiary grade A class hospitals in Beijing, China. Patients who died or were discharged within the first 24 h after admission were excluded. The study protocol was approved by the ethics committee for clinical research in our hospital before the performance of the analyses (code: 2017-P2–124-01).

### Demographic data

All demographic data were collected from medical records, including age, gender, length of hospital stay (LOS), and medical history. According to previous statistics, the two major causes of mortality among people aged 85 years and above are cardiovascular disease (including ischemic heart disease, cerebrovascular disease, hypertension and diabetes) and cancer [17]. For each participant, we collected data for 12 common chronic diseases including hypertension, myocardial infarction, atrial fibrillation, cerebrovascular diseases, malignant diseases (including solid tumor and hematological malignancy), diabetes mellitus or impaired glucose tolerance, chronic obstructive pulmonary disease, thyroid dysfunction (including hyperthyroidism and hypothyroidism), dementia, peptic ulcers, anxiety or depression state, and chronic kidney disease of stage 3b or worse (estimated glomerular filtration rate [eGFR]-EPI < 45 ml/min) [18]. Patients with more than one of the above diseases were considered as having multi-morbidity. We recorded the use of antibiotics on admission.

### Functional status and clinical information

The functional status of each patient approximately 1 week before hospitalization was assessed on admission by the trained nurses. The Barthel Index (BI) was used to assess the functional status for activities of daily living (ADL) in each participant [19]. This scale includes 10 items, such as eating, bathing, grooming, dressing, defecation, urination, going to the toilet, getting out of bed, walking, and stair-climbing. Each item is 0,5,10,15 points respectively. The ADL function of the patient is reflected by the sum of scores for all of the items. A maximum score of 100 indicates that the subject is independent in physical function and the lowest score of 0 indicates a totally dependent state. We divided the Barthel Index score by 5, and we got 21 categories, from 0 to 20 ADL category. Patients were divided into two groups according to the BI scores: severe disability and full dependency (BI: 0–45) or mild/moderate disability and independency (BI: 50–100) [20].

Blood biochemical indexes including creatinine, glucose, glycated albumin, albumin, total triglyceride, total cholesterol, uric acid, potassium, sodium, hypersensitivity C reactive protein, white blood cell count, platelet count, and hemoglobin were routinely examined on the next morning after an overnight fast of at least 8 h. We used the CKD-EPI-SCr formula to calculate the eGFR. Hypoalbuminemia was defined by a serum albumin level < 35 g/l [21]. Anemia was diagnosed based on the criterion of a hemoglobin level < 110 g/l in females and < 120 g/l in males [22].

### Statistical analyses

Statistical analysis was performed using SPSS 16.0 software (SPSS Inc., Chicago, IL, USA). Categorical variables were expressed as numbers (percentages) and analyzed using the chi-square test or Fisher’s Exact Test. Normally distributed continuous variables were expressed as the mean ± standard deviation (SD), with comparisons between groups made using the independent t-test. Non-normally distributed continuous variables were expressed as median (interquartile range), and the Wilcoxon rank sum test was used. Forward Selection [Likelihood Ratio (LR)] in binary logistic regression analysis was used to determine the independent factors associated with the incidence of in-hospital death (a_in_ = 0.05, a_out_ = 0.10). All variables that were significant on univariate analysis were entered in the subsequent multivariate analysis. Adjusted odds ratios (ORs) with 95% confidence intervals (CIs) were calculated. A *p* value < 0.05 was considered statistically significant.

## Results

### Patient characteristics

For patients who had been hospitalized more than once, the most recent medical records from multiple admissions were analyzed in this study. Overall, a total of 162 patients were included. The median age of the patients was 92 years, and 72.8% were male. During the hospitalization, 46 patients died, and the others were discharged. The site/type of infection was acute respiratory infection in 141 patients, digestive system infection in 13 patients, urinary tract infection in 6 patients, and skin infection in 2 patients. The three most common comorbidities were hypertension (*n* = 127, 78.4%), diabetes mellitus or impaired glucose tolerance (*n* = 80, 49.4%), and chronic obstructive pulmonary disease (*n* = 66, 40.7%). Multimorbidity was very common in this cohort (*n* = 144, 88.9%), but there was no significant difference between the two groups. Compared with those who were discharged, patients who died during hospitalization were older, more likely to have atrial fibrillation, and more likely to have a history of malignant disease (Table 1). According to the predefined diagnostic criteria, 70 patients (43.2%) were classified as having severe disability and full dependency, and 70 patients (43.2%) had hypoalbuminemia. Seventy-nine patients (48.8%) were diagnosed with anemia.

**Table 1:** Baseline characteristics in nonagenarian patients hospitalized for acute infectious diseases

	Total (*n* = 162)	Died group (*n* = 46)	Discharged group (*n* = 116)	χ^2^/Z	*P* value
Age [years, M (IQR)]	92.0 (91.0,95.0)	93.0 (92.0,95.0)	92.0 (91.0,94.0)	−2.598	0.009
Male (%)	118 (72.8)	36 (78.3)	82 (70.7)	0.954	0.329
LOS [days, M (IQR)]	27.0 (14.0,45.3)	33.5 (12.0, 71.3)	25 (14.3, 38.0)	−1.750	0.080
MD (%)	43 (26.5)	19 (41.3)	24 (20.7)	7.179	0.007
AF (%)	52 (32.1)	21 (45.7)	31 (26.7)	5.414	0.020
HT (%)	127 (78.4)	33 (71.7)	94 (81.0)	1.680	0.195
OMI (%)	24 (14.8)	9 (19.6)	15 (12.9)	1.149	0.284
DM/IGT (%)	80 (49.4)	20 (43.5)	60 (51.7)	0.896	0.344
COPD (%)	66 (40.7)	15 (32.6)	51 (44.0)	1.760	0.185
CKD (%)	41 (25.3)	14 (30.4)	27 (23.3)	0.893	0.345
CVD (%)	51 (31.5)	15 (32.6)	36 (31.0)	0.038	0.846
Dementia (%)	35 (21.6)	12 (26.1)	23 (19.8)	0.762	0.383
TD (%)	19 (11.7)	5 (10.9)	14 (12.1)	0.046	0.831
Anxiety/Depression (%)	24 (14.8)	7 (15.2)	17 (14.7)	0.008	0.928
PU (%)	15 (9.3)	6 (13.0)	9 (7.8)	1.095	0.295
Multimorbidity (%)	144 (88.9)	41 (89.1)	103 (88.8)	0.004	0.951
Antibiotic use (%)^a^	156 (96.3)	45 (97.8)	111 (95.7)	0.422	0.516

*M* median; *IQR* interquartile range; *LOS* (length of hospital stay); *MD* malignant disease; *AF* atrial fibrillation; *HT* hypertension; *OMI* old myocardial infarction; *DM/IGT* diabetes or impaired glucose tolerance; *COPD* chronic obstructive pulmonary disease; *CKD* chronic kidney disease 3b or worse; *CVD* cerebrovascular disease; *TD* thyroid dysfunction; *PU* peptic ulcers

^a^: analyzed by Fisher’s Exact Test

### Predictors of in-hospital mortality

The results of univariate analysis indicated that patients who died during hospitalization had a significantly lower BI, lower serum hemoglobin and albumin levels, and a higher serum glucose level and white blood cell count than those who were discharged (Table 2). All variables that were significant in the univariate analysis entered in the multivariate analysis. Forward selection [likelihood ratio (LR)] in binary logistic regression analysis revealed that malignant disease (OR 2.73, 95%CI 1.10–6.78), ADL category (OR 0.82, 95%CI 0.75–0.89), and serum albumin (OR 0.86, 95%CI 0.78–0.95) were independent risk factors for in-hospital mortality among nonagenarian patients with infectious diseases (Table 3).

**Table 2:** Baseline functional status and blood biochemical indexes in nonagenarian patients hospitalized for acute infectious diseases

	Total (*n* = 162)	Died group (*n* = 46)	Discharged group (*n* = 116)	t/Z	*P* value
ADL category (score, $\overline{x}$ ±s)	10.01 ± 5.49	5.96 ± 4.66	11.62 ± 4.95	−6.671	< 0.001
WBC (× 10^9^/L, $\overline{x}$ ±s)	8.59 ± 4.35	10.15 ± 5.44	7.98 ± 3.68	2.493	0.015
HGB (g/L, $\overline{x}$ ±s)	115.04 ± 19.15	107.94 ± 22.09	117.86 ± 17.16	−3.046	0.003
ALB (g/L, $\overline{x}$ ±s)	35.24 ± 4.37	32.42 ± 4.43	36.36 ± 3.83	−5.656	< 0.001
GLU [mmol/l, M (IQR)]	5.61 (4.88,6.86)	6.33 (5.15,8.73)	5.56 (4.82,6.37)	−2.271	0.023
PLT (× 10^12^/L, $\overline{x}$ ±s)	187.04 ± 77.37	197.04 ± 107.06	183.08 ± 61.96	1.036	0.302
e-GRF [ml/min, M(Q1,Q3)]	62.08 (44.32,76.26)	64.13 (37.79,76.43)	61.11 (45.67,76.25)	−0.470	0.638
UA (μmol/l, $\overline{x}$ ±s)	314.55 ± 119.76	321.41 ± 147.67	311.83 ± 107.33	0.400	0.690
GA (%, $\overline{x}$ ±s)	17.09 ± 3.27	17.40 ± 3.92	16.97 ± 2.98	0.751	0.454
TC (mmol/l, $\overline{x}$ ±s)	3.65 ± 0.82	3.45 ± 0.91	3.72 ± 0.77	−1.911	0.058
TG [mmol/l, M (IQR)]	0.84 (0.62,1.11)	0.83 (0.70,1.03)	0.86 (0.60,1.22)	−0.013	0.990
K [mmol/l, M (IQR)]	4.00 (3.78,4.33)	3.92 (3.72,4.39)	4.01 (3.79,4.32)	−0.141	0.888
NA (mmol/l, $\overline{x}$ ±s)	137.90 ± 5.63	138.35 ± 6.69	137.72 ± 5.17	0.639	0.524
Hs-CRP [mg/l, M(Q1,Q3)]	21.72 (6.98,37.77)	28.47 (10.04,46.81)	19.10 (6.64,37.32)	−1.639	0.101

*ADL* activities of daily living; *WBC* white blood cell count; *HGB* hemoglobin; *PLT* platelet count; *eGFR* estimated glomerular filtration rate; *ALB* albumin; *UA* uric acid; *GLU* glucose; *GA* glycated albumin; *TC* total cholesterol; *TG* triglyceride; *K* potassium; *NA* sodium; *Hs-CRP* hypersensitivity C reactive protein

**Table 3:** Predictors of in-hospital mortality in nonagenarians with acute infection: results of likelihood ratio forward selection in multivariate logistic regression analyses

Variables	Odds ratio	95% confidence interval	*P* value
ADL category	0.82	0.75–0.89	< 0.001
ALB	0.86	0.78–0.95	0.004
MD	2.73	1.10–6.78	0.030

*ADL* activities of daily living; *ALB* albumin; *MD* malignant disease

Preceding factors included in the model included: AGE, MD, AF, ADL category, WBC,ALB, HGB, GLU

## Discussion

In this retrospective cohort study, we found that the in-hospital mortality for nonagenarian patients admitted for acute infection was quite high (17.2%). Moreover, functional impairment as well as serum albumin were independent predictors of in-hospital mortality in our cohort of nonagenarian patients hospitalized for acute infectious diseases. These findings suggest that it is very necessary to stratify the patients based on BI score and serum albumin. Additionally, maintaining good functional status and supplementation with albumin may be important for improving clinical outcomes in nonagenarian patients hospitalized for acute infectious diseases.

Previous studies confirmed that, independent of the type of disease diagnosed on admission, the in-hospital mortality rate among nonagenarian patients is much higher than that among patients 65–90 years of age [23]. Similarly, the in-hospital mortality rate was 17.2% in our study, with one in six patients aged 90 years or older suffering from acute infectious diseases died in our study. This is consistent with previous studies in which the in-hospital mortality for the nonagenarian population ranged from 13.3–22.8% in internal medicine departments and geriatric acute units [15, 16, 23–25]. With regard to the sites of infection, a previous report in Taiwan showed that pneumonia and urinary tract infection were the two major infectious diseases on admission for nonagenarians [26]. Similarly, respiratory tract infection was the main cause of acute infection in our cohort, highlighting the importance of the prevention of respiratory tract infection in these patients. Moreover, we found that the prevalence of atrial fibrillation and malignant diseases differed significantly between those who discharged and those died during the hospitalization. The presence of malignant diseases results in a more than 2-fold increase in the likelihood of in-hospital death in multivariate analysis. A previous research in Israel also found that atrial fibrillation and malignant diseases were the main predictors of in-hospital mortality among nonagenarians [15], which is similar to our findings. Previous epidemiological data from China showed that the prevalence of atrial fibrillation in the general population aged ≥60 years was 1.83% in China [27], which further confirmed the increased prevalence of atrial fibrillation with aging. Notably, although the proportion of patients with multimorbidity in our cohort was high (144/162, 88.9%), the proportions of patients with multimorbidity did not differ statistically between two groups. This is different from previous findings, which showed that multimorbidity was associated with a higher mortality rate in a population-based study of nonagenarians [28, 29] and in hospitalized nonagenarians [16, 24]. However, a previous study showed that geriatric conditions rather than the multimorbidity predicted the risk of mortality in an octogenarian population [30]. These inconsistencies may be explained by the different management statuses of the diseases in the octogenarian patients in different studies. Nonagenarian patients may have good prognosis if their diseases are well treated, despite the presence of multimorbidity.

We found that functional impairment as well as serum albumin were independent predictors of in-hospital mortality in our cohort of nonagenarian patients hospitalized for acute infectious diseases. For every 5-point increase in the BI score, the likelihood of in-hospital death decreased by 18%. For every 1-unit increase in serum albumin, the odds of death decreased by 14%. ADL is one of the most important factors that reflect the health status of an individual. Poor ADL at discharge has been associated with increased risk of 1-year mortality for older patients undergoing percutaneous coronary intervention [31]. Serum albumin plays a vital physiologic role in health maintenance for many organs. Hypoalbuminemia had been historically linked to patients’ nutritional status, and recently, it has been suggested that hypoalbuminemia is an inflammatory marker rather than an index of malnutrition in sarcoidosis patients [32]. Hypoalbuminemia also increases the short-term mortality for patients attending the emergency department, and the long-term mortality is also increased in older patients with hypoalbuminemia [33, 34]. Taken together, our results also confirmed the clinical prognostic properties of ADL and serum albumin for mortality in population-based or hospitalized nonagenarians [16, 17, 25, 28, 35, 36]. A previous study showed that for nonagenarian patients, due to acute medical illnesses, hypoalbuminemia and functional loss at the time of discharge are factors associated with 1-year mortality [25]. In the Rugao longevity cohort in China, both serum albumin and ADL were effective predictors of all-cause mortality in long-lived individuals aged 95 years or older, and the combination of albumin and ADL was recommended as an inexpensive, easy-to-use screening method for risk stratification of nonagenarian patients [35]. Stratification of patients on admission, maintenance of good functional status, and supplementation with albumin may be important for improving clinical outcomes in nonagenarian patients hospitalized for acute infectious diseases. Prospective clinical trials are needed to confirm it.

There are limitations in this study. First, this was a retrospective single-center study. The results of our study should be confirmed in prospective trials. Second, other types of comprehensive geriatric assessments besides functional status were not performed in our study, such as delirium assessment, cognitive assessment, and visual or hearing impairment assessment. The association between these factors and in-hospital mortality in nonagenarian patients hospitalized for acute infectious disease remains to be determined. Finally, the sample size of the cohort was relatively small, that makes the extension and generalization of the results difficult. The predictive efficacies of functional status and hypoalbuminemia for mortality risk after discharge should be evaluated in the future.

## Conclusions

In conclusion, in this study nonagenarian patients with acute infectious diseases admitted to the internal medical department had high in-hospital mortality, with one in six dying during hospitalization. Serum albumin and poor functional status on admission were independent predictors of in-hospital mortality in our cohort of nonagenarian patients hospitalized for acute infectious diseases.

## Data Availability

The datasets used during the current study are available from the corresponding author on reasonable request.

## Abbreviations

ADL: Activities of daily living
AF: Atrial fibrillation
ALB: Albumin
CKD: Chronic kidney disease 3b or worse
CKD-EPI-SCr: Chronic Kidney Disease Epidemiology Collaboration serum creatinine
COPD: Chronic obstructive pulmonary disease
CVD: Cerebrovascular disease
DM/IGT: Diabetes or impaired glucose tolerance
eGFR: Estimated glomerular filtration rate
GA: Glycated albumin
GLU: Glucose
HGB: Hemoglobin
hs-CRP: Hypersensitivity C reactive protein
HT: Hypertension
IQR: Interquartile range
K: Potassium
LOS: Length of hospital stay
M: Median
MD: Malignant disease
NA: Sodium
OMI: Old myocardial infarction
PLT: Platelet count
PU: Peptic ulcers
TC: Total cholesterol
TD: Thyroid dysfunction
TG: Triglyceride
UA: Uric acid
WBC: White blood cell count.

## References

[1] Robine JM, Paccaud F (2005). Nonagenarians and centenarians in Switzerland, 1860-2001: a demographic analysis. J Epidemiol Community Health.

[2] National Data. National Bureau of statistics of People’s republic of China. 2019. http://data.stats.gov.cn/. Accessed 20 Mar 2019.

[3] Wang L, Li Y, Liu C, Yang Y, Chen Y, Yang H (2015). Risk factors for mortality in nonagenarians with femoral neck fractures undergoing joint replacement. Zhonghua Yi Xue Za Zhi.

[4] Hayes AJ, Davda A, El-Hadi M, Murphy P, Papettas T (2016). Short and medium-term outcomes for general surgery in nonagenarian patients in a district general hospital. Ann R Coll Surg Engl.

[5] Fariña-Castro R, Roque-Castellano C, Marchena-Gómez J, Rodríguez-Pérez A (2017). Five-year survival after surgery in nonagenarian patients. Geriatr Gerontol Int.

[6] Ovidiu A, Stefan GT, Dragos P, Bogdan V, Dana AI (2017). Survival of nonagenarian patients with hip fractures: a cohort study. Acta Ortop Bras.

[7] Uehara K, Matsuda H, Inoue Y, Omura A, Seike Y, Sasaki H, et al. Is Conventional Open Repair for Abdominal Aortic Aneurysm Feasible in Nonagenarians? Ann Vasc Dis. 2017;10(3). 10.3400/avd.oa.17-00013. 10.3400/avd.oa.17-00013 PMC5684160 29147161

[8] Rivoirard R, Chargari C, Kullab S, Trone JC, Langrand-Escure J, Moriceau G (2016). Chemotherapy regimen in nonagenarian Cancer patients: a bi-institutional experience. Chemotherapy.

[9] Kim JY, Jeong MH, Choi YW, Ahn YK, Chae SC, Hur SH (2015). Temporal trends and in-hospital outcomes of primary percutaneous coronary intervention in nonagenarians with ST-segment elevation myocardial infarction. Korean J Intern Med.

[10] Mizuguchi Y, Hashimoto S, Yamada T, Taniguchi N, Nakajima S, Hata T (2016). Percutaneous coronary intervention for nonagenarian patients with ST-segment elevation myocardial infarction: experience of a single Japanese center. J Cardiol.

[11] Behrouz R, Masjuán-Vallejo J, Vera R, Willey JZ, Zedet M, Moulin S (2018). Outcomes of nonagenarians with acute ischemic stroke treated with intravenous Thrombolytics. J Stroke Cerebrovasc Dis.

[12] Sahin M, Ocal L, Kalkan AK, Kilicgedik A, Kalkan ME, Teymen B (2017). In-hospital and long term results of primary angioplasty and medical therapy in nonagenarian patients with acute myocardial infarction. J Cardiovasc Thorac Res.

[13] Hwabejire JO, Kaafarani HM, Lee J, Yeh DD, Fagenholz P, King DR (2014). Patterns of injury, outcomes, and predictors of in-hospital and 1-year mortality in nonagenarian and centenarian trauma patients. JAMA Surg.

[14] Ramos JM, Sánchez-Martínez R, Nieto F, Sastre J, Valero B, Priego M (2013). Characteristics and outcome in nonagenarians admitted in general internal medicine and other specialties. Eur J Intern Med.

[15] Zafrir B, Laor A, Bitterman H (2010). Nonagenarians in internal medicine: characteristics, outcomes and predictors for in-hospital and post-discharge mortality. Isr Med Assoc J.

[16] Conde-Martel A, Hemmersbach-Miller M, Marchena-Gomez J, Saavedra-Santana P, Betancor-Leon P (2012). Five-year survival and prognostic factors in a cohort of hospitalized nonagenarians. Eur J Intern Med.

[17] Rivoirard R, Chargari C, Trone JC, Falk AT, Guy JB, Eddekaoui H (2014). General management of nonagenarian patients: a review of the literature. Swiss Med Wkly.

[18] Gansevoort RT, de Jong PE (2010). Challenges for the present CKD classification system. Curr Opin Nephrol Hypertens.

[19] Mahoney FI, Barthel DW (1965). Functional evaluation: the Barthel index. Md State Med J.

[20] Gatta A, Verardo A, Bolognesi M (2012). Hypoalbuminemia. Intern Emerg Med.

[21] Shah S, Vanclay F, Cooper B (1989). Improving the sensitivity of the Barthel index for stroke rehabilitation. J Clin Epidemiol.

[22] Lu Z, Zhong N (2011). Internal Medicine.

[23] Barba R, Martínez JM, Zapatero A, Plaza S, Losa JE, Canora J (2011). Mortality and complications in very old patients (90+) admitted to departments of internal medicine in Spain. Eur J Intern Med.

[24] Singer M, Conde-Martel A, Hemmersbach-Miller M, Ruiz-Hernández JJ, Arencibia Borrego J, Alonso OB (2018). Mortality hospital of nonagenarian patients in internal medicine. Rev Clin Esp.

[25] Socorro García A, de la Puente M, Perdomo B, López Pardo P, Baztán JJ (2015). Functional status and mortality at month and year in nonagenarians hospitalized due to acute medical illness. Eur J Intern Med.

[26] Chao CT, Lin YF, Tsai HB, Hsu NC, Tseng CL, Ko WJ, HINT Study Group (2013). In nonagenarians, acute kidney injury predicts in-hospital mortality, while heart failure predicts hospital length of stay. PLoS One.

[27] Li Y, Wu YF, Chen KP, Li X, Zhang X, Xie GQ (2013). Prevalence of atrial fibrillation in China and its risk factors. Biomed Environ Sci.

[28] Formiga F, Ferrer A, Pérez-Castejón J, Riera-Mestre A, Chivite D, Pujol R (2009). Mortality-associated factors in nonagenarian. The Nona-Santfeliu study. Two years of follow-up. Rev Clin Esp.

[29] Formiga F, Ferrer A, Chivite D, Rubio-Rivas M, Cuerpo S, Pujol. R (2011). Predictors of long-term survival in nonagenarians: the NonaSantfeliu study. Age Ageing.

[30] Lu FP, Chang WC, Wu SC (2016). Geriatric conditions, rather than multimorbidity, as predictors of disability and mortality among octogenarians: a population-based cohort study. Geriatr Gerontol Int.

[31] Higuchi S, Kabeya Y, Matsushita K, Taguchi H, Ishiguro H, Kohshoh H (2016). Barthel index as a predictor of 1-year mortality in very elderly patients who underwent percutaneous coronary intervention for acute coronary syndrome: better activities of daily living. Longer Life Clin Cardiol.

[32] Mirsaeidi M, Omar HR, Sweiss N (2018). Hypoalbuminemia is related to inflammation rather than malnutrition in sarcoidosis. Eur J Intern Med.

[33] Lyons O, Whelan B, Bennett K, O'Riordan D, Silke B (2010). Serum albumin as an outcome predictor in hospital emergency medical admissions. Eur J Intern Med.

[34] Corona LP, de Oliveira Duarte YA, Lebrão ML (2018). Markers of nutritional status and mortality in older adults: the role of anemia and hypoalbuminemia. Geriatr Gerontol Int.

[35] Liu Z, Zhong G, Li S, Deng W, Zhang Y, Qian D (2015). Use of serum albumin and activities of daily living to predict mortality in long-lived individuals over 95 years of age: a population-based study. Age (Dordr).

[36] Yust-Katz S, Katz-Leurer M, Katz L, Lerman Y, Slutzki K, Ohry A (2005). Characteristics and outcomes of ninth and tenth decade patients hospitalized in a sub-acute geriatric hospital. Isr Med Assoc J.

